# Maternal nicotinic exposure produces a depressed hypoxic ventilatory response and subsequent death in postnatal rats

**DOI:** 10.14814/phy2.12023

**Published:** 2014-05-28

**Authors:** Jianguo Zhuang, Lei Zhao, Fadi Xu

**Affiliations:** 1 Pathophysiology Program, Lovelace Respiratory Research Institute, Albuquerque, New Mexico

**Keywords:** Cardiorespiratory failure, cotinine, hypoxemia, maternal cigarette smoking, sudden infant death syndrome

## Abstract

In this study, we asked whether a “full term” prenatal nicotinic exposure (fPNE, 6 mg·kg^−1^·day^−1^ nicotinic delivery) over the full gestation, compared to a traditional PNE (tPNE) over the last two‐thirds of the gestation, caused a higher mortality following a remarkable depressed hypoxic ventilatory response (dHVR) independent of brain and pulmonary edema and change in serum corticosterone. P12‐14 pups pretreated with tPNE, fPNE or their vehicle (tCtrl and fCtrl) were exposed to 5% O_2_ for up to 60 min followed by harvesting the brain and lungs or anesthetized to collect blood for detecting arterial blood pH/gases and serum cotinine and corticosterone levels. We found that fPNE had little effect on baseline *V*_E_ and heart rate, but consistently induced a dHVR and prolonged apnea that were rarely observed after tPNE. The severity of the dHVR in PNE pups were closely correlated to an earlier appearance of lethal ventilatory arrest (the hypoxia‐induced mortality). PNE did not induce brain and pulmonary edema, but significantly increased serum corticosterone levels similarly in tPNE and fPNE pups. Moreover, the accumulated nicotinic dose given to the individual was significantly higher in fPNE than tPNE pups, though there was no difference in serum cotinine levels and arterial blood pH/gases between the two groups. Our results suggest that nicotinic exposure at the early stage of gestation achieved by fPNE, rather than tPNE, is critical in generating the dHVR and subsequent death occurring independently of brain/pulmonary edema and changes in arterial blood pH/gases and serum corticosterone.

## Abstract (precis)

Our results suggest that nicotinic exposure at the early stage of gestation achieved by “full term” prenatal nicotinic exposure (fPNE), rather than traditional prenatal nicotinic exposure (tPNE), is critical in generating the depressed hypoxic ventilatory response (dHVR) and subsequent death. The fPNE‐induced cardiorespiratory impairement is independent of brain/pulmonary edema and changes in arterial blood pH/gases and serum corticosterone.

## Introduction

Sudden infant death syndrome (SIDS) is a condition in which cardiorespiratory failure associated with hypoxemia occurs during sleep (Hunt and Brouillette 1987; Kinney and Filiano 1988; Kandall and Gaines 1991). Maternal cigarette smoke during pregnancy is one of the highest risk factors for SIDS, presumably through activation of nicotinic receptors (Duncan et al. 2008, 2009; Slotkin and Seidler 2011).

Because SIDS occurs in seemingly healthy infants, it is difficult to determine its pathogenesis. SIDS has long been hypothesized to result from impaired cardiorespiratory responses to acute hypoxia induced by sleep apnea or rebreathing of exhaled gas in the prone sleep position (Hunt 1992; Poets et al. 1993). In support, a depressed hypoxic ventilatory response (dHVR) has been observed in the near‐miss SIDS victims (Hunt et al. 1981; Wennergren et al. 1983) and the infants born to cigarette‐smoking mothers (Ueda et al. 1999; Parslow et al. 2003, 2004). However, the relationship between the dHVR and SIDS has not been established by analysis of their correlation without interferences from other factors (apparent life‐threatening events, family members, and prematurity) related to SIDS (Hall and Zalman 2005).

Effects of prenatal nicotinic exposure (PNE) on hypoxic ventilatory response (HVR) and hypoxia‐induced death have also been investigated in animals. Among these studies, traditional PNE (tPNE) is achieved by subcutaneously delivering nicotine (6 mg·kg^−1^·day^−1^) via an osmotic minipump, usually over the last two‐thirds of the 21‐day gestation in rats. This pretreatment leads to (1) an excessive mortality (15%) during 5% O_2_ for 60 min (Slotkin et al. 1995); (2) an aggravated apneic/gasping response to hypoxia with little change in baseline minute ventilation (*V*_E_), heart rate (HR), or metabolism (Bamford et al. 1996; Fewell et al. 2001; Robinson et al. 2002); and (3) a mildly blunted (Eugenin et al. 2008) or unchanged HVR (Bamford et al. 1996; Bamford and Carroll 1999; Robinson et al. 2002) in neonates after birth. We postulated that the lower mortality and lack of a constant dHVR in these studies were due to a limited nicotinic exposure. Nicotinic delivery in these studies was usually started on gestational days 6–7 after the embryo was implanted in the uterine wall (Slotkin et al. 1995). In sharp contrast, maternal cigarette smoking in humans commonly starts before rather than during pregnancy. About 20–25% of women in the United States keep smoking during pregnancy despite extensive warnings about adverse impact of smoking on the fetus (Chiolero et al. 2005). To date, no study has been carried out to assess the effects of “full term” PNE (fPNE) over the gestation on the offspring's blood nicotinic levels, cardiorespiratory activities, and hypoxia‐induced cardiorespiratory response and mortality.

In addition to functional changes as mentioned above, investigators have linked the presence of brain and pulmonary edema in some SIDS victims to the cardiorespiratory failure (Aoki 1994; Krous et al. 2007). Furthermore, an increased volume of the brain was reported in some SIDS cases (O'Kusky et al. 1995; Kadhim et al. 2005). Cigarette smoke could raise blood corticosterone level in a dose‐dependent manner (Andersson et al. 1985) and the elevated corticosterone in rat pups is able to affect respiration and HVR (Gulemetova and Kinkead 2011). Thus, it is important to determine whether fPNE is able to induce the dHVR associated with brain and pulmonary edema (brain volume change) and change in corticosterone levels in rat pups.

## Materials and Methods

Thirteen male and 26 female pathogen‐free Sprague–Dawley rats (250–350 g) were purchased from Charles River Laboratories, Inc. (Wilmington, MA); housed in the animal facility at Lovelace Respiratory Research Institute in filter top cages; and provided with water and food ad libitum. The room was constantly ventilated and the temperature was kept at 23°C. The animals were quarantined for 2 weeks before experiments. The experimental protocols were conducted in accordance with the Guide for the Care and Use of Laboratory Animals and approved by the Institutional Animal Care and Use Committee, which is accredited by the Association for Assessment and Accreditation of Laboratory Animal Care International, USA.

### Pretreatment with PNE

The females were randomly designated to receive tPNE (*n* = 8) and fPNE pretreatment (*n* = 8) and their vehicle, tCtrl (*n* = 5) and fCtrl (*n* = 5), respectively. The dams and their offspring used in this study are summarized in Table 1. The fCtrl and fPNE pretreatment were achieved as previously reported except for an extended exposure period (Slotkin et al. 1995). Briefly, animals were sufficiently anesthetized by 2–5% isoflurane coupled with a local anesthetic (Bupivicaine, 0.25 mg·kg^−1^). An appropriately sized incision was made on the shoulder area of the dorsal back to permit insertion of an osmotic minipump (2.5 *μ*L·h^−1^ for 28 days, Alza Corp., Palo Alto, CA), followed by closing the wound under sterile surgical conditions. Therefore, the females were continuously exposed to vehicle or nicotine tartrate (6 mg·kg^−1^·day^−1^). The latter was reported to produce nicotine blood levels approximately equivalent to or higher than that observed in moderate to heavy smokers (Slotkin et al. 1997; Hussein et al. 2007). Analgesic (Metacam suspension, 0.2 mg·kg^−1^, oral) was administered 30 min before the end of the surgery and Q12 h (prn) for 1–2 days postsurgery to prevent discomfort. The animals recovered in their home cages. Ten days after the surgery, each female rat was placed in a breeding cage with a male rat for up to 4 days. The females with vaginal plugs were considered pregnant and separated from the male. They were anesthetized again on the seventh day of gestation to replace the minipump with a new one filled with vehicle (fCtrl) or the same nicotine dose for fPNE. The tCtrl and tPNE were prepared in the same manner with the exception that the females only received the minipump implantation once on the seventh day of gestation.

**Table 1.** The numbers of dams and their pups used in this study

Pups' group	I‐pup#	II‐pup#	I + II‐pup#	Dams#
tCtrl	7	7	14	5
fCtrl	8	7	15	5
tPNE	15	8	23	8
fPNE	16	8	24	8
Total	46	30	76	26

fPNE, “full term” prenatal nicotinic exposure; tPNE, traditional prenatal nicotinic exposure.

I and II represent Study Series I and II. # = animal numbers. No more than three male pups from each litter were used to minimize the possible effect of genetic difference between litters on the results.

### Animal grouping

Rat pups born by spontaneous vaginal delivery were housed with their mother and siblings (24–25°C, and 12:12 h light/dark cycle). In all experiments, no more than three male pups from each litter with similar overall litter size were used to minimize the possible effect of genetic difference between litters on the results. Males were chosen in this study because males are much more vulnerable than females in human SIDS (Adams et al. 1998). Rat pups were utilized in two study series (Table 1); one for measuring Vco_2_, *V*_E_, HR followed by analysis of edema in the brain and lungs; and another for detecting arterial blood pH and gases, serum cotinine and corticosterone levels (detailed below).

### Habituation to the two chambers used for determining Vco_2_ and ventilation respectively

In Study Series I, four groups of male rat pups at P10‐12 (postnatal day 10–day 12) were individually placed in a 60 mL syringe chamber (with the plunger removed) for 10 min. The animal was then moved out from the syringe chamber and placed into a whole‐body unrestrained plethysmograph chamber (PLY3211; Buxco Electronics Inc., Troy, NY) with a bias flow (0.5 L·min^−1^) for ~70 min. The same habituation was applied once a day for three continuous days.

### Measurements of metabolism, *V*_E_ and heart rate

After habituation in both chambers, Vco_2_ was first measured in pups at P12‐14. The pups' brain development at this period is equivalent to newborn infants at 2–4 months (Ballanyi 2004). As reported before (Liu et al. 2009), the individual pup was placed in the syringe chamber and its open end was closed by a plug with an inlet connected to a flow regulator (Bias flow regulator; Buxco Research Systems, Wilmington, NC). CO_2_ concentration (by using a CO_2_ analyzer, Hewlett Packard 78356A), temperature, and humidity in the air out of the syringe were continuously measured to determine Vco_2_. Two needle ECG electrodes were placed in the nape of the neck in each animal after local anesthesia (bupivacaine, s.c.). The animal was then placed in the plethysmograph with flexible thin wires from ECG electrodes exiting through the plethysmograph's outlet followed by sealing. The ECG signals were amplified with a bio‐amplifier (ML135; ADInstruments Inc., Colorado Springs, CO).The plethysmograph was continuously flushed with normoxic (21% O_2_ and 79% N_2_) gas mixtures at 0.5 L·min^−1^ that did not evoke any consistent change in breathing, indicating no mechanical interference from the gases puff with the animal's breathing. Hypoxic challenge was administered by switching the normoxic to a hypoxic gas mixture (5% O_2_ balance with N_2_). The Buxco plethysmograph was reported to have considerable and insurmountable problems for measuring tidal volume (*V*_T_) in small animals, including rat pups (Mortola and Frappell 1998). However, this should not be an issue in this study because the fPNE‐induced dHVR is the result of significant reduction in respiratory frequency (*f*_R_) rather than *V*_T_ (see Figs. 2, 3). The temperature inside the chamber was maintained at ~30.0°C as reported before (Pendlebury et al. 2008; Boychuk et al. 2011) through adjusting a heating lamp outside of the chamber, by which the animal body temperature (BT) was maintained at ~36.5°C. Calibrations for flow rate and gas concentrations were made before and after each experiment. All studies were performed during 9:00 and 17:00 h to avoid any influence from the circadian rhythm (Stephenson et al. 2001).

### Lung and brain water contents (the brain volume)

Following completion of hypoxic exposure (see below), the animal was euthanatized with urethane (2.4 g·kg^−1^, ip) and the brain and lungs were harvested. The mean volume of the brain was measured as previously described (Siebert and Haas 1994; O'Kusky et al. 1995; Kadhim et al. 2005). Subsequently, samples of lung and brain tissues were weighed by an electronic balance. The wet sample was dried in an isotemperature oven (Model NO. 97‐920‐1; Fisher Scientific Inc., Pittsburgh, PA) at 60°C for 48 or 72 h. The tissues were weighed once every day after drying in the oven until the final two weights of the tissues became the same, and this weight was defined as dry weight of the tissue. The water content of the tissue was calculated as the dry/wet ratio to assess the pulmonary and brain edema.

### Blood sample collections

Four additional groups of pups at P12‐14 (Study Series II) were anesthetized with urethane (1200 mg·kg^−1^, ip). As needed, supplemental urethane (300 mg·kg^−1^, ip) was administered to completely eliminate eye‐blink and limb‐withdrawal reflex. The right femoral artery was isolated and cannulated, and arterial blood was sampled (130 *μ*L) for measurements of baseline pH and blood gases. After the animal was euthanized (Euthasol 150 mg·kg^−1^, ip), 0.5–0.6 mL venous blood was withdrawn from the right ventricle. Subsequently, the blood sample was centrifuged (15,000 *g*, 4°C for 5 min) and serum was collected and placed in a −80°C freezer for later analysis of serum cotinine and corticosterone levels.

### Measurements of pH and blood gases

The baseline pH and blood gases in anesthetized pups were determined by using a blood gases analyzer (GEM Premier 3000; Instrumentation Lab., Lexington, MA).

### Cotinine detection

Cotinine is the primary metabolite of nicotine that accurately reflects nicotine intake with a relatively long half‐life (Bordia et al. 2008). Thus, the exposure of pups to nicotine was ascertained by measuring their serum cotinine. The latter was detected with a Cotinine Direct ELISA kit (CalBiotech, Spring Valley, CA) following the manufacturer's instructions as previously reported (Hapidin et al. 2007).

### Corticosterone measurement

Analysis of corticosterone was performed using an ELISA kit (ab108821; Abcam, Cambridge, MA) and a microplate spectrophotometer (*μ*‐Quant; Bio‐Tek Instruments, Winooski, VT) as previously described (Gulemetova and Kinkead 2011). Corticosterone concentrations were calculated from the parameters of the standard curve linearized by a log–log transformation.

### Experimental protocols

In Study Series I, body weight (BW) of tCtrl, fCtrl, tPNE, and fPNE P12‐14 pups (*n* = 7, 8, 15, and 16, respectively) was weighed. After measuring baseline Vco_2_, the animals instrumented with ECG leads were individually placed in the plethysmograph and rectal temperature was measured by a thermistor (ADInstruments Inc.). The pups were exposed to normoxia for 15–20 min followed by hypoxia (5% O_2_ balance with N_2_) for up to 60 min. The pups surviving after 60 min hypoxia and those showing a cessation of HR for 30 sec during hypoxia were immediately euthanized followed by harvesting the lungs and brain. In Study Series II, tCtrl, fCtrl, tPNE, and fPNE P12‐14 pups (*n* = 7, 7, 8, and 8, respectively) were anesthetized under room air for collection of arterial blood.

### Data acquisition and statistical analysis

Raw data of the airflow, ECG, CO_2_ concentrations, and temperature were digitized, monitored, and recorded by PowerLab/8sp (model ML 785; ADInstruments Inc.) and a computer with the LabChart Pro 7 software. Respiratory variables including *V*_T_, *f*_R_, and minute ventilation (*V*_E_) were derived by the online calculations of the airflow signals. HR was derived from each inter‐beat (R‐R) interval of the ECG signal. BT, BW, blood gases and pH, dry/wet ratio of lung and brain tissues, the brain volume, serum cotinine, and corticosterone levels were analyzed. All variables were expressed as absolute values with the exception that cardiorespiratory response to hypoxia was presented as Δ% change from the baseline values unless mentioned otherwise. The baseline values were determined by measuring the variables for 1 min immediately before hypoxia. Cardiorespiratory response to hypoxia was measured during the initial HVR and subsequent HVR. We measured the former for 1 min at the period 2–4 min after hypoxia (with the peak *V*_E_ response) and the latter for 5 min at the period 25–30 min after hypoxia. It should be noted that apnea usually occurred 30 min after hypoxia. A T_E_ equal to or longer than 2 sec was defined as an apnea as pointed out in the previous studies (Xu et al. 2003; Pendlebury et al. 2008). A rapid inspiratory rise with a prolonged expiratory phase was defined as gasping (Poets et al. 1999; Sridhar et al. 2003). Group data were reported as means ± SE. Two‐way analysis of variance (ANOVA) with repeated measures was used to analyze the significant differences among the four groups. If an overall test was significant, Tukey's test was utilized for specific comparisons between individual groups. Comparisons of mortality among the four groups were performed with Fisher's exact probability test followed by multiple comparisons using Bonferroni's test. *P*‐values <0.05 were considered significant.

## Results

### PNE does not cause significant behavior changes

The pregnant rats undergoing either fPNE or tPNE had no discernible behavior abnormalities, such as agitation, loss of appetite, or shortness of breath. All pups in the four groups were delivered vaginally at full term of gestational day 21 without dead fetuses found. There was no significant difference in birth numbers among tCtrl, fCtrl, tPNE, and fPNE groups (10.3 ± 1.3 vs. 10.6 ± 1.1 vs. 9.8 ± 0.8 vs. 10.5 ± 1.0; *P* > 0.05).

### PNE increases serum cotinine without effect on baseline metabolism, cardiorespiratory activities, and plasma corticosterone level

**Figure 1.**
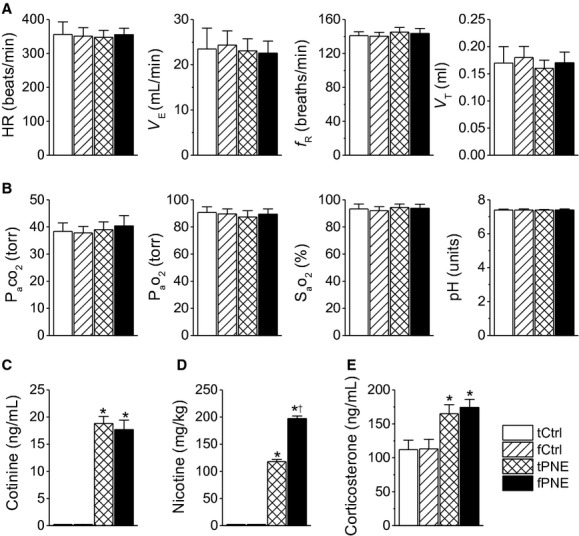
Comparison of cardiorespiratory activities (A), arterial blood pH/gases (B), serum cotinine levels (C), accumulated nicotine delivered from the minipump (D), and serum corticosterone levels (E) among tCtrl, fCtrl, traditional prenatal nicotinic exposure (tPNE), and fPNE pups. Data presented in panel (A) were obtained from Study Series I (*n* = 7, 8, 15, and 16 for tCtrl, fCtrl, tPNE, and fPNE pups, respectively), while those in panels (B, C, D, and E) from Study Series II (*n* = 7, 7, 8, and 8 for tCtrl, fCtrl, tPNE, and fPNE pups, respectively). Mean ± SE. *P < 0.01 compared to the Ctrls, and ^†^*P* < 0.01 compared to tPNE. HR, heart rate; *V*_E_, minute ventilation, *f*_R_, respiratory frequency; *V*_T_, tidal volume; P_a_o_2_ and P_a_co_2_, partial pressures of arterial blood oxygen and carbon dioxide; S_a_o_2_, oxygen saturation in arterial blood.

We compared BW, BT, and Vco_2_ among the four groups of pups. As presented in Table 2, both fPNE and tPNE did not significantly alter the animals' BW, BT, or Vco_2_ at P12‐14. Moreover, neither fPNE nor tPNE strikingly changed baseline *V*_E_ and HR (Fig. 1A) or blood pH and gases (Fig. 1B). Serum cotinine was undetectable in both Ctrls, but it was profoundly increased after PNE with no difference between the two PNE groups (Fig. 1C). The nicotinic concentration and dose (2.5 *μ*L·h^−1^, 6 mg·kg^−1^·day^−1^) delivered daily in this study were similar to the previous tPNE (Slotkin et al. 1995; Bamford et al. 1996; Fewell et al. 2001; Eugenin et al. 2008). However, the accumulated total nicotinic exposure was strikingly higher in the fPNE than tPNE individual due to the advance and prolongation of PNE in the former (Fig. 1D). Serum corticosterone was markedly increased by PNE without a difference between the two treated groups (Fig. 1E).

**Table 2.** BW, BT, and Vco_2_ in four groups of pups

	tCtrl (*n* = 7)	fCtrl (*n* = 8)	tPNE (*n* = 15)	fPNE (*n* = 16)
BW (g)	29.3 ± 3.0	28.7 ± 2.3	30.2 ± 3.5	32.9 ± 3.5
BT (°C)	36.6 ± 0.07	36.8 ± 0.07	36.5 ± 0.08	36.3 ± 0.09
Vco_2_ (mL·min^−1^·kg^−1^, STPD)	44.3 ± 1.8	45.3 ± 1.2	44.7 ± 1.4	44.1 ± 1.6

BW, body weight; BT, body temperature; fPNE, “full term” prenatal nicotinic exposure; tPNE, traditional prenatal nicotinic exposure; STPD, standard temperature, and pressure, dry air.

### fPNE, compared to tPNE, leads to a more severe dHVR and a higher mortality

**Figure 2.**
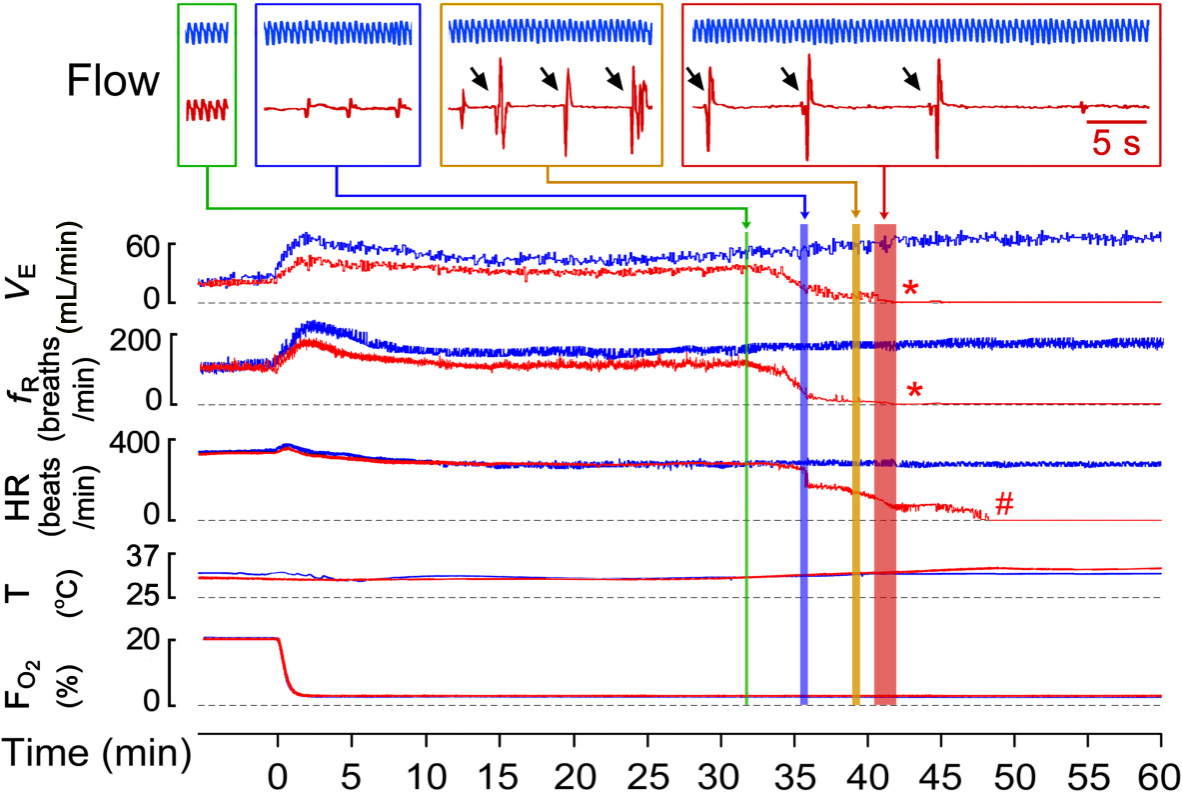
Typical recordings of cardiorespiratory responses to 5% O_2_ for 60 min in an fPNE (red) and a traditional prenatal nicotinic exposure (tPNE; blue) pup (Ctrls = tPNE, not shown). Flow, insets of representative airflow signals that sequentially show baseline ventilation, apneas, gasps following the apnea, and the lethal ventilatory arrest after gasps. The gasps are pointed by arrows. *V*_E_, minute ventilation; *f*_R_, respiratory frequency; HR, heart rate; T, temperature in the chamber; and Fo_2_, O_2_ fraction in the plethysmographic chamber. Hypoxia starts at time “0” and * and # represent the lethal *V*_E_ arrest and the termination of heart beat, respectively.

We determined the cardiorespiratory responses to hypoxia (5% O_2_ for up to 60 min) in the four groups of pups. The typical recordings of the cardiorespiratory responses to hypoxia obtained from an fPNE and a tPNE pup are illustrated in Figure 2. Compared to tPNE, fPNE produced a remarkable dHVR. Hypoxia for ~35 min evoked apneas and then gasps in the fPNE, but usually not in the tPNE pups, leading to a lethal ventilatory arrest (death) several minutes later. Cardiac failure appeared several minutes following the lethal ventilatory arrest. In general, the PNE pups who showed a severe dHVR (>25%↓ of Ctrl HVR) died during the hypoxia (*n* = 10 for fPNE and 2 for tPNE), while those who presented mild dHVR (<25%↓ of Ctrl HVR) survived (*n* = 6 for fPNE and 13 for tPNE).

**Figure 3.**
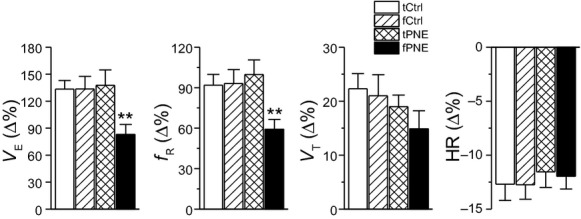
Group data showing the responses of minute ventilation (*V*_E_), respiratory frequency (*f*_R_), tidal volume (*V*_T_), and heart rate (HR) at the 30th min of hypoxia (5% O_2_) in tCtrl, fCtrl, tPNE, and fPNE pups (*n* = 7, 8, 15, and 16, respectively). Mean ± SE. All data are significantly (*P* < 0.01) different from the corresponding baseline values (“0”). **P < 0.01 compared to the Ctrls and tPNE pups.

**Figure 4.**
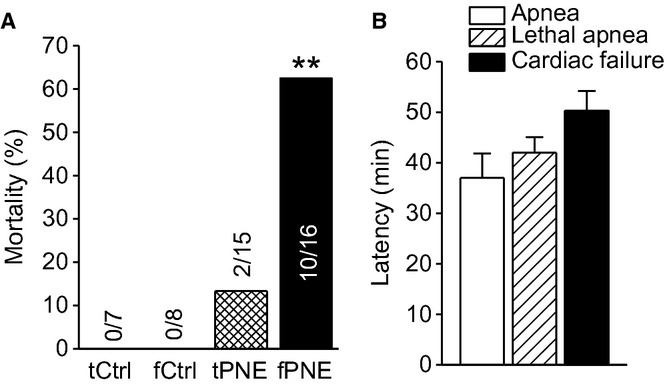
(A) Mortality in the tCtrl, fCtrl, traditional prenatal nicotinic exposure (tPNE), and fPNE pups (*n* = 7, 8, 15, and 16, respectively). (B) The latency required for generating the hypoxia‐induced apnea, lethal ventilatory arrest, and cardiac failure in the dead PNE pups (*n* = 12). Mean ± SE, **P < 0.01 compared to the Ctrl and tPNE pups.

Statistically, during the initial hypoxia, fPNE pups presented a significant reduction in HVR (↓38%) predominantly due to a smaller *f*_R_ response without remarkable changes in *V*_T_ and HR responses (Fig. 3). During the subsequent hypoxia, a significantly lower HVR (34%) was observed in fPNE rather than tPNE pups. In addition, bradycardia occurred at this period in tCtrl, fCtrl, tPNE, and fPNE pups (12 ± 2.6%, 13 ± 2.1%, 13 ± 1.8%, and 14 ± 2.3%, *P* < 0.01 compared to the data before hypoxia) without differences among the groups. Interestingly, bradycardia became much worse in fPNE, but not tPNE and Ctrls, pups during 35–40 min after hypoxia (45 ± 5.1% for fPNE vs. 13 ± 2.2%, 13 ± 1.9%, and 14 ± 2.4% for tCtrl, fCtrl, and tPNE, *P* < 0.01), leading to a cardiac arrest followed by death. As depicted in Figure 4A, hypoxia‐induced mortality was significantly higher in the fPNE (63%) than the tPNE pups (13%) with no death in both Ctrl groups. Hypoxia‐induced apneic and gasping responses in all nonsurviving pups. The latency of the first apnea, lethal arrest, and cardiac failure (heart cessation) in dead pups (*n* = 12 including 10 for fPNE and 2 for tPNE) were summarized in Figure 4B. After breakdown, we found that nonsurviving fPNE and tPNE pups did not show remarkable differences in their apneic numbers (26 ± 5 for fPNE vs. 22 and 25 for tPNE) and the onset of the first apnea (36 ± 5 min for fPNE vs. 33 and 37 min for tPNE). However, the averaged apneic duration was longer in fPNE than tPNE pups (4.6 ± 0.8 sec for fPNE vs. 3.2 and 3.5 sec for tPNE). Importantly, the appearance of the lethal ventilatory arrest appeared earlier in 10 dead fPNE pups (42 ± 2 min after hypoxia) than two dead tPNE pups (48 and 59 min after hypoxia).

### The severity of dHVR is correlated to the death and appearing time of the lethal ventilatory arrest

**Figure 5.**
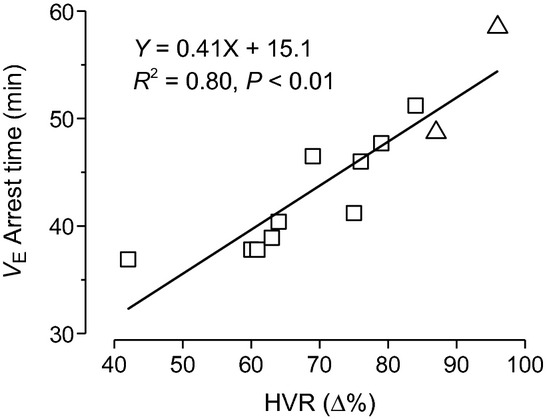
Correlation between the severity of depressed hypoxic ventilatory response (dHVR; the lower HVR) and the apparent time of the lethal ventilatory arrest in 10 fPNE (□) and two traditional prenatal nicotinic exposure (tPNE) pups (Δ). X and Y represent dHVR and *V*_E_ arrest time, respectively.

As mentioned above, when a given PNE‐pretreated pup showed a remarkable dHVR (>25%↓ of the Ctrl HVR), the pup died eventually during 60 min hypoxia, which clearly demonstrates a correlation between the PNE‐induced severity of dHVR and the mortality. We further correlated the degree of dHVR to the appearing time of the lethal ventilatory arrest during hypoxia (*n* = 10 and 2 in fPNE and tPNE pups). As exhibited in Figure 5, the pups presenting more severe dHVR (lower HVR) often displayed the ventilatory arrest earlier. In other words, the fPNE pups with more severe dHVR (lower HVR) likely died earlier during the hypoxia.

### fPNE changes neither lung/brain water contents nor the brain volume

**Figure 6.**
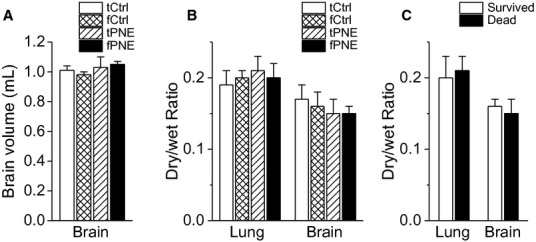
Effect of PNE on lung and brain tissues. Panels (A and B) summarize the brain volume and dry/wet ratio of lung and brain tissues among tCtrl, fCtrl, traditional prenatal nicotinic exposure (tPNE), and fPNE groups (*n* = 7, 8, 15, and 16), respectively. Panel (C) compares dry/wet ratio of lung and brain tissues between 34 surviving (*n* = 7, 8, 13, and 6 for tCtrl, fCtrl, tPNE, and fPNE pups) and 12 dead pups (*n* = 10 and 2 for fPNE and tPNE) during hypoxic exposure. Mean ± SE.

We compared the brain volume and found that the values were not significantly different among the four groups of pups (Fig. 6A). By comparing dry/wet ratio of lung and brain tissues, we also found that there were no significant differences in dry/wet weight ratios of lung and brain tissues among the four groups (Fig. 6B). To delineate whether hypoxia‐induced pulmonary and/or brain edema contributing to the death, we further analyzed dry/wet ratio of lung and brain tissues between the 34 pups surviving after hypoxia (*n* = 7, 8, 13, and 6 for tCtrl, fCtrl, tPNE, and fPNE pups) and 12 pups that died due to hypoxia (*n* = 10 and 2 for fPNE and tPNE). As illustrated in Figure 6C, both ratios were not significantly different between the surviving and dead pups.

## Discussion

One of our novel findings in this study was that fPNE significantly depresses the initial HVR by 38% and the subsequent HVR by 34%, which is significantly different from a mild (<20% reduction; Eugenin et al. 2008) or no change in HVR, (Bamford et al. 1996; Bamford and Carroll 1999; Robinson et al. 2002) previously reported in tPNE pups. The dHVR in this study was not associated with changes in baseline *V*_E_, HR, blood gases, and Vco_2_, consistent with the previous reports showing a lack of these changes in tPNE pups (Bamford et al. 1996; Fewell et al. 2001; Robinson et al. 2002). It is generally accepted that the initial HVR (peak response) is predominately mediated by activation of peripheral chemoreceptors, while the subsequent HVR decline reflects the sum of the hypoxic stimulation of peripheral chemoreceptor and hypoxic inhibition of the central nervous system (CNS). Clearly, the reduction in the initial HVR observed in this study suggests that fPNE is able to suppress the peripheral chemoreceptor‐mediated HVR. However, we cannot distinguish whether there is a stronger CNS inhibition in fPNE than tPNE pups to join in the worsened subsequent HVR. We measured serum cotinine levels in P12‐14 pups and found little difference between fPNE and tPNE pups, which is in line with the report that prolongation of the osmotic pumping did not change the maximal nicotinic concentration in maternal serum (Fewell et al. 2001). Our finding suggests that insufficient PNE in the early phase of gestation may be responsible for the lack of a constant appearance of dHVR in tPNE pups. In other words, the nicotinic exposure at the early stage of gestation is critical in developing the dHVR and subsequently death in our animal model. One may concern that fPNE exerts its influence on ventilation via altering embryonic implantation in the uterine wall or blood flow without effect on epigenetic modification on fetal nerve function. However, this concern is strongly argued by our recent results showing an ability of fPNE to increase bronchopulmonary C fiber‐mediated apneic response and overexpress the density of their fibers' expression (Zhuang et al. 2014). It remains unknown which cells' replication, differentiation, growth/death, and/or gene expressions are uniquely affected by fPNE, rather than tPNE, and responsible for the dHVR (mortality).

Another important finding in this study is that fPNE, as compared to tPNE, induces a higher mortality in response to hypoxia following more severe dHVR. Although the dHVR is assumed to contribute to the SIDS in the clinic (Ueda et al. 1999; Harris and St‐John 2005), this correlation has not been established in SIDS victims without interferences from apparent life‐threatening events, family members, and prematurity that are related to SIDS (Hall and Zalman 2005). Our results, for the first time, not only reveal the more powerful role fPNE plays, as compared to tPNE, in inducing the respiratory failure during hypoxia, but also demonstrate a close correlation between the severity of dHVR and death independent of apparent life‐threatening events, family members, and prematurity. Collectively, our data point to a possible contribution of the dHVR to the respiratory failure, but studies are warranted to define the extent to which the dHVR is causative to the respiratory failure. The possible mechanisms underlying the fPNE‐induced dHVR and aggravation of apneic response remain unclear. The fact that the dHVR results from a depressed *f*_R_ response in this study, similar to a depressed *f*_R_ response to hypoxia observed in infants with prenatal cigarette smoking (Schneider et al. 2008), favors an involvement of abnormal neural control of breathing. In support, there was no difference in blood pH/gases and corticosterone levels between fPNE and tPNE pups (Fig. 1). How does fPNE affect the neurons responsible for respiratory rhythmic response to hypoxia? Peripherally, nicotine exposure was reported to reduce dopamine content (a stimulating neurotransmitter) in the carotid bodies (Holgert et al. 1995). In addition, nicotinic cholinergic receptors exist in the fetal nerve system and play an important role in neural maturation (Navarro et al. 1989). PNE over the gestation may lead to a remarkable perturbation of neural maturation, such as the neural immaturity characterized by overexpression of vagal C‐fibers in SIDS victims (Becker et al. 1993). Vagal C‐fibers, especially its pulmonary C‐fibers (PCFs), are inhibitory to HVR and critical in generating central apnea and lethal ventilatory arrest during pathophysiological conditions (Xu et al. 2003). These results lead to an assumption that fPNE may be able to blunt peripheral chemosensitivity and/or increase PCF density/sensitization, contributing to the respiratory failure. Centrally, SIDS is reportedly associated with central 5‐HT deficiency (Kinney et al. 2003) and this deficiency could induce apnea and death in rat pups exposed to several episodes of environmental anoxia (Cummings et al. 2011). Moreover, PNE could impair the presynaptic release of both GABA and glutamate to produce breathing disorders, including central apnea (Fregosi and Pilarski 2008). These findings point to other possible explanations for the fPNE‐induced respiratory failure. Ultimately, further studies are certainly needed to elucidate the mechanisms underlying the fPNE‐induced respiratory failure.

We asked whether tPNE and fPNE could produce brain and pulmonary edema in this study. As presented in Figure 6, the brain volume and dry/wet ratio of the brain and lung were not significantly different among the four groups and between the surviving and dead pups after hypoxia. These data differ from some victims of SIDS showing a brain volume change or pulmonary/brain edema (Aoki 1994; O'Kusky et al. 1995; Kadhim et al. 2005; Krous et al. 2007), but are similar to other SIDS victims who have no such changes (Berry 1992; Falck and Rajs 1995; O'Kusky et al. 1995). One of the explanations for this discrepancy is that there are two different subgroups of SIDS victims with or without these brain and pulmonary abnormalities in the clinic, and our animal model fits the latter.

Our results showed that severe hypoxia, but not PNE, induced apneic responses. More importantly, the hypoxia caused a longer apnea and an earlier appearance of the lethal ventilatory arrest in fPNE than tPNE pups. This suggests a role of fPNE in exaggerating the hypoxia‐induced respiratory failure. Consistent with our findings, it was reported that a near‐miss SIDS victim had no apnea during normoxia, periodic breathing (prolonged expiration) during mild hypoxia (17% O_2_), and apneic episodes (even lethal ventilatory arrest) during severe hypoxia (Wennergren et al. 1983). Furthermore, a recent report shows that the hypoxia‐induced apnea is more severe in PNE than Ctrl newborn mice (Robinson et al. 2002). With respect to the cardiac responses, our results demonstrated that (1) fPNE induced a severe bradycardia 35–40 min after hypoxia; (2) the bradycardia appeared immediately following apnea; and (3) the lethal ventilatory arrest occurred before cardiac arrest. Consistent with our findings, it has been reported that PNE is capable of decreasing HR response to hypoxia (Slotkin et al. 1997; Hafstrom et al. 2002b) in a dose‐dependent manner in animals (Hafstrom et al. 2004). Moreover, remarkable bradycardia associated with apnea was observed in SIDS victims (Poets et al. 1999) and rat pups exposed to multiple episodes of environmental anoxia (97% N_2_, 3% CO_2_; Fewell et al. 2001; Cummings et al. 2011). Although it is debatable, central apnea could occur prior to bradycardia in some SIDS victims (Poets et al. 1999). We believe that fPNE is capable of suppressing HVR and aggravating apnea to worsen hypoxemia during acute hypoxia, and that the interaction of fPNE and severe hypoxemia consequently impair cardiorespiratory functions, leading to death.

There are several limitations in this study. First, pulmonary inflammation is observed in 20% of SIDS victims (Blood‐Siegfried et al. 2002, 2004; Prandota 2004; Vege and Ole Rognum 2004; Kinney and Thach 2009) and upregulation of inflammatory mediators, such as IL‐1*β* and substance P, is assumed to be responsible for SIDS (Jordan et al. 1997; Froen et al. 2000; Balan et al. 2011). Although pulmonary edema is absent in our model, our data cannot deny the presence of pulmonary inflammation. Second, we cannot rule out a possible damage of pulmonary function induced by fPNE that may be involved in the respiratory failure observed in this study. Recent evidence has shown an increase in airway resistance or appearance of airway obstruction in SIDS infants (Kato et al. 2001; Byard and Krous 2003; Thach 2005) or animal models (Hafstrom et al. 2002a). Third, in this study we did not implant a sham osmotic pump in tPNE pups at the time when the first minipump was implanted in the fPNE. Thus, we cannot exclude the potential contribution of the absence of this sham implantation to the little effect of tPNE on the respiratory failure even though it is unlikely. Fourth, nicotine is one of the major toxic components of cigarette smoking, thus PNE may not reflect full toxicity of maternal cigarette smoke in generating SIDS. However, it is noteworthy that the close relationship between the fPNE‐induced dHVR and the respiratory failure in this study supports the belief that PNE plays an important role in generating SIDS.

### Perspectives and significance

Sudden infant death syndrome usually occurs in children 2–4 months old and is the third leading cause of infant mortality with about 2500 deaths per year in the United States (http://www.sids.org/). Maternal cigarette smoking that includes nicotinic exposure highly correlates with SIDS, however, the mechanisms underlying the pathogenesis of SIDS remain unclear. Our results show a severe and consistent dHVR in fPNE, but not tPNE, pups closely correlated with the mortality, suggesting that fPNE is a more suitable animal model relevant to SIDS. The fact that the severe and consistent dHVR is only observed after fPNE further demonstrates a critical impact of cigarette smoking at early stage of gestation on developing the dHVR and respiratory failure. In conclusion, our results not only benefit our understanding of nicotinic toxicology in control of cardiorespiratory activities, but also to gain insight into the susceptibility of exposure to cigarette smoking in utero in developing SIDS.

## Conflict of Interest

None declared.
